# Genetically engineered rice endogenous 5-enolpyruvoylshikimate-3-phosphate synthase (*epsps*) transgene alters phenology and fitness of crop-wild hybrid offspring

**DOI:** 10.1038/s41598-017-07089-9

**Published:** 2017-07-28

**Authors:** Xiao Yang, Lei Li, Xiaoqi Jiang, Wei Wang, Xingxing Cai, Jun Su, Feng Wang, Bao-Rong Lu

**Affiliations:** 1 Ministry of Education Key Laboratory for Biodiversity and Ecological Engineering, Institute of Biodiversity Science, Fudan University, Songhu Road 2005, Shanghai, 200438 China; 2 Fujian Province Key Laboratory of Genetic Engineering for Agriculture, Fujian Academy of Agricultural Sciences, Fuzhou, 350003 China

## Abstract

Genetically engineered (GE) rice endogenous *epsps* (5-enolpyruvoylshikimate-3-phosphate synthase) gene overexpressing EPSPS can increase glyphosate herbicide-resistance of cultivated rice. This type of *epsps* transgene can enhance the fecundity of rice crop-weed hybrid offspring in the absence of glyphosate, stimulating great concerns over undesired environmental impacts of transgene flow to populations of wild relatives. Here, we report the substantial alteration of phenology and fitness traits in F_1_-F_3_ crop-wild hybrid descendants derived from crosses between an *epsps* GE rice line and two endangered wild rice (*Oryza rufipogon*) populations, based on the common-garden field experiments. Under the glyphosate-free condition, transgenic hybrid lineages showed significantly earlier tillering and flowering, as well as increased fecundity and overwintering survival/regeneration abilities. In addition, a negative correlation was observed between the contents of endogenous EPSPS of wild, weedy, and cultivated rice parents and fitness differences caused by the incorporation of the *epsps* transgene. Namely, a lower level of endogenous EPSPS in the transgene-recipient populations displayed a more pronounced enhancement in fitness. The altered phenology and enhanced fitness of crop-wild hybrid offspring by the *epsps* transgene may cause unwanted environmental consequences when this type of glyphosate-resistance transgene introgressed into wild rice populations through gene flow.

## Introduction

The unpredicted potential environmental impact caused by transgene flow from genetically engineered (GE) crops to their cross-compatible wild relatives has stimulated tremendous debates and studies over the last decades^1–3^. Wild relative populations that have acquired a strongly fitted transgene through gene flow likely changes their evolutionary potential, resulting in unwanted environmental/ecological consequences^4–7^. It is therefore essential to properly assess the environmental impact of transgene flow before the commercialization of any GE crops. One of the key points to assess such environmental impact is to determine fitness of a transgene introgressed into wild populations^7, 8^, provided that the frequency of crop-to-wild gene flow is known. Many studies have been carried out to determine the fitness effect of a transgene under the controlled field-experimental conditions, involving crop-wild hybrid lineages. These studies included hybrid descendants derived from crosses between squash-wild gourd^9, 10^, maize-teosinte^11^, cultivated-wild sunflowers^12^, and cultivated-wild/weedy rice^13–18^. The generated data provided useful information for the biosafety assessment of environmental impact caused by transgene flow.

Herbicide-resistant GE crops are predominantly cultivated in the world because of their evident advantages to reduce the crop production costs through effective agriculture weed control^19, 20^. To date, GE crops containing an herbicide-resistance transgene(s) have occupied nearly 90% of the global arable lands where all GE crops with different transgenes are grown, including GE soybean, cotton, maize, and canola^21^. Breeders are enthusiastic to develop herbicide-resistant rice (*Oryza sativa* L.)—a very important world cereal crops, to meet the challenge of world food security by resolving the great problems of weed control, particularly under the situation of shifting rice cultivation from traditional transplantation to direct seeding^22, 23^. In direct-seeding fields, rice production faces more serious problems for weed control, particularly for weedy rice (*O*. *sativa* f. *spontanea*, referred to as WDR hereafter) that belongs to the same biological species of cultivated rice^24, 25^. Supposedly, the application of herbicide-resistant rice can effectively reduce the infestation of weedy rice^23, 26^, although this proposal faces challenges^16, 20, 23^. That is, the introgression of herbicide-resistance transgenes from GE rice into WDR populations through spontaneous gene flow and its social/environmental impact by increased weed problems becomes a great biosafety concern^3^. For WDR that mostly infests rice fields, the rapid spread of an introgressed herbicide-resistance transgene to its populations is expected because of the strong selective pressure from herbicide application in rice fields. WDR populations with substantially increased herbicide-resistance and weediness have been found in Clearfield^®^ rice fields, causing tremendous weed problems^27, 28^. The concerns over the spread of herbicide-resistance transgenes to WDR populations is reasonable in the development and commercialization of herbicide-resistant GE rice.

However, it is argued that the spread of herbicide-resistance transgenes to wild rice (*Oryza* species) populations may not cause similar problems as in WDR populations, because wild rice populations occur in nature habitat where no herbicides are used and therefore selection pressure from herbicides does not exist^29, 30^. In contrast, herbicide-resistance transgenes are considered to be neutral or even have costs in plants under the herbicide-free environmental conditions^31, 32^. Therefore, wild rice populations that have acquired an herbicide-resistance transgene through gene flow may not have any benefit due to the lack of selective pressure from herbicides^33^. Nevertheless, a recent study reported significantly increased fecundity and ratios of tryptophan concentration and photosynthesis in crop-weed hybrid lineages containing an herbicide-resistance transgene, at the absence of herbicide^16^. This transgene is an engineered rice endogenous *epsps* (5-enolpyruvoylshikimate-3-phosphate synthase) gene that overexpresses EPSPS. The GE rice was originally developed to confer tolerance to the glyphosate herbicide^34^, but unexpectedly the *epsps* transgene provided substantial benefits to WDR for plant growth and seed production^16^. This phenomenon poses a question about the transgene that overproduces EPSPS. Will the *epsps* herbicide-resistance transgene introgressed into any of the wild rice species produce the same fitness effect to their populations?

The perennial common wild rice (*O*. *rufipogon* Griff., referred to as WR hereafter) is the direct ancestor of Asian cultivated rice and one of the wild species in the genus *Oryza* (Poaceae). WR is distributed in the tropics and subtropics of monsoon Asia, with its northernmost border in Jiangxi province of China^35^. WR can reproduce sexually through seeds, or asexually through propagule or ratooning^36, 37^. It is widely recognized that WR is important germplasm for the genetic improvement of cultivated rice. For example, the well-known hybrid rice breeding program was benefited from the discovery and use of a male sterility (*ms*) gene from WR^38, 39^, demonstrating the importance of germplasm in WR gene pool^40^. However, WR is under threats due to the rapid growth of human population and urbanization, dramatic change in agriculture land uses, and intensive human disturbances^41, 42^. Also, massive and continued introgression of cultivated rice genes and transgenes into WR have posed a great challenge on the existence of WR populations^3, 43^. Altogether, identified gene flow from cultivated rice to WR populations in the controlled experiments^44, 45^ and population genetic studies^46^ indicated the high probability of transgene introgression from GE rice to WR. In addition, the diverse genetic variability among WR populations may result in different fitness responses of WR recipients to the same transgene, as revealed in a recent study of crop-weed hybrids containing insect-resistance transgenes^18^.

We produced F_1_-F_3_ crop-wild isogenic hybrid lineages with or without the *epsps* transgene, derived from artificial crosses between an *epsps* GE rice line and two WR populations. The objectives of this study were to address the following questions. (1) Does the over-expressing *epsps* transgene change the life-cycle traits of crop-wild hybrid descendants in the glyphosate-free environment? (2) Does the *epsps* transgene increase the fecundity and over-winter survival of crop-wild hybrid descendants? (3) Does the genetic background of transgene recipients affect the fitness of the *epsps* transgene in different types of rice parents, including WR, WDR, and cultivated rice? The answer of these questions will help us to appropriately estimate the potential environmental/ecological consequences caused by introgression of the overexpressing *epsps* herbicide-resistance transgene into WR populations, likely also to predict the potential consequences for other crop-wild transgene introgressions.

## Results

### More tillers and earlier flowering with increased seed sets in transgenic hybrid lineages

**Figure 1:**
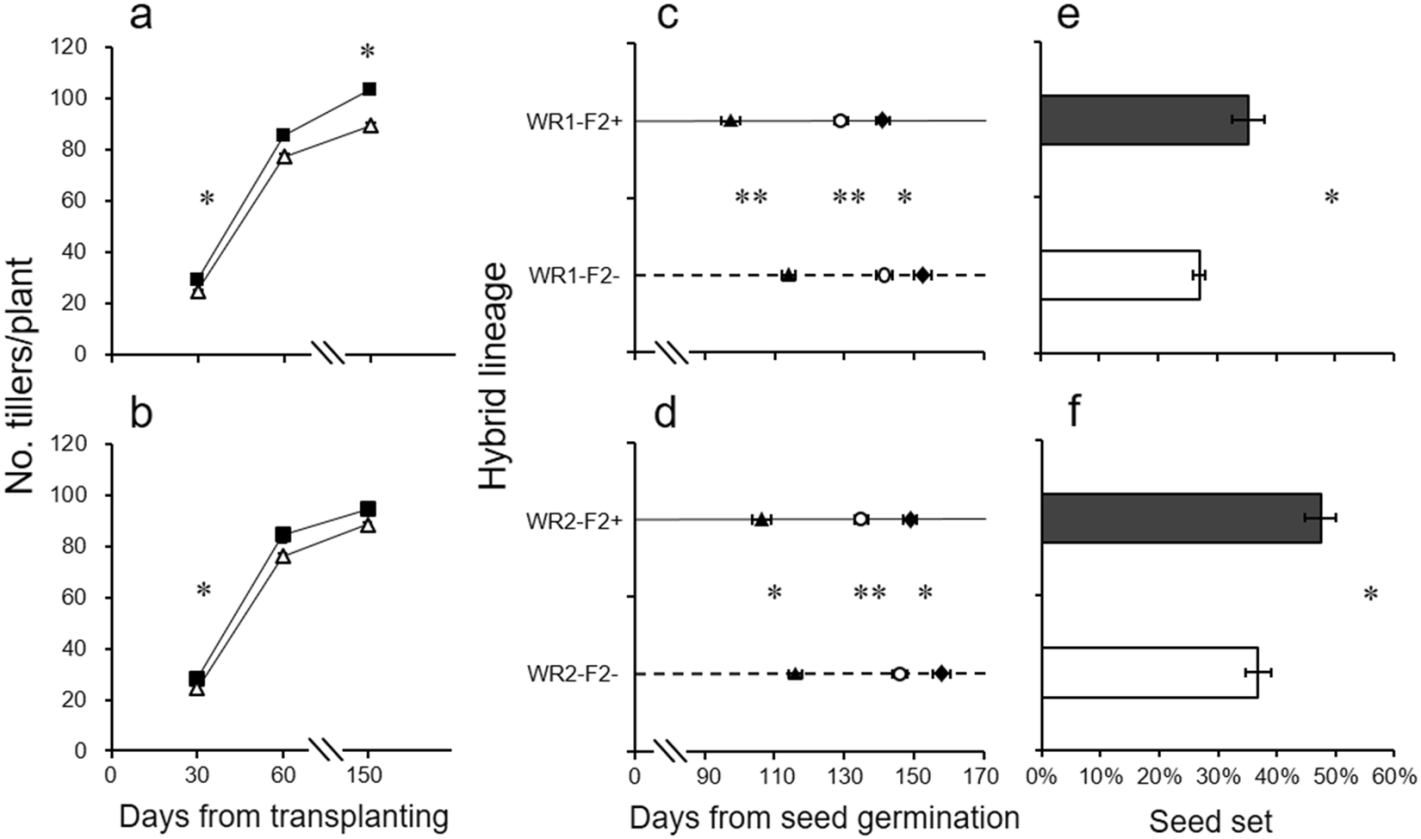
Number of tillers per plant (**a**: WR1-F2; **b**: WR2-F2); flowering time (**c**: WR1-F2; **d**: WR2-F2); and seed set ratios (**e**: WR1-F2; f: WR2-F2) of F_2_ transgenic (solid squares, dark grey columns) and non-transgenic (empty triangles, white columns) hybrid lineages in pure planting. Solid triangles, empty circles, and solid diamonds (in c and d) indicate days at which 1%, 30%, and 50% plants being flowered, respectively. The comparisons were made between transgenic and non-transgenic F_2_ hybrid lineages in pure-planting based on independent *t*-test (N = 6). Bars represent standard error. * or ** indicates significances at the levels of P < 0.05 or P < 0.01, respectively.

F_1_ and F_2_ hybrid lineages with the *epsps* transgene showed a greater number of tillers and earlier flowering time than their isogenic controls without the transgene (Fig. 1a, b, c and d; Tables S1–S3). Consequently, ratios of seed sets in the transgenic hybrid lineages showed significant increase, compared to their non-transgenic counterparts (Fig. 1e and f; Table S1). An obvious negative correlation was observed between ratio of seed set and days from seed germination to flowering in F_2_ hybrid descendants (Fig. S1a and b). In addition, no differences were observed for plant height between transgenic and non-transgenic hybrid lineages (Table 1).

**Table 1:** Two-way ANOVAs for the effects of transgene (transgenic *vs*. non-transgenic), wild parent (WR1 *vs*. WR2), and their interactions on life-cycle fitness related traits of F_1_-F_3_ rice crop-wild hybrid descendants.

Trait	Transgene (T)			Wild parent (WR)			T × WR		
	df	F	P	df	F	P	df	F	P
F_1_ experiment									
Plant height	1	0.076	0.786	1	0.011	0.916	1	0.158	0.695
No. tillers per plant-30 days	1	22.293	0.000	1	12.107	0.002	1	0.957	0.340
No. tillers per plant-60 days	1	22.212	0.000	1	0.053	0.820	1	0.385	0.542
No. tillers per plant-150 days	1	26.674	0.000	1	0.064	0.803	1	0.463	0.504
Days for 1% plants to flower	1	7.347	0.013	1	1.837	0.190	1	0.000	1.000
Days for 30% plants to flower	1	6.792	0.017	1	6.792	0.017	1	0.189	0.669
Days for 50% plants to flower	1	5.902	0.025	1	13.279	0.002	1	0.000	1.000
No. panicles per plant	1	9.205	0.007	1	0.720	0.406	1	0.208	0.654
No. seeds per plant	1	3.541	0.074	1	12.544	0.002	1	0.367	0.551
1000-seed weight	1	0.779	0.388	1	0.819	0.376	1	0.667	0.424
Ratio of seed set	1	5.760	0.026	1	33.180	0.000	1	3.687	0.069
Ratio of tiller regeneration	1	14.861	0.000	1	0.763	0.384	1	2.213	0.140
F_2_ experiment									
Plant height	1	0.592	0.451	1	5.910	0.025	1	0.407	0.531
No. tillers per plant-30 days	1	12.593	0.002	1	0.161	0.693	1	0.040	0.844
No. tillers per plant-60 days	1	3.278	0.085	1	0.055	0.816	1	0.001	0.976
No. tillers per plant-150 days	1	9.428	0.006	1	2.077	0.165	1	1.498	0.235
Days for 1% plants to flower	1	23.719	0.000	1	4.246	0.053	1	1.719	0.205
Days for 30% plants to flower	1	25.508	0.000	1	5.092	0.035	1	0.104	0.751
Days for 50% plants to flower	1	17.474	0.000	1	7.578	0.012	1	0.260	0.616
No. panicles per plant	1	7.822	0.011	1	0.018	0.894	1	0.202	0.658
No. seeds per plant	1	9.171	0.007	1	46.538	0.000	1	0.067	0.799
1000-seed weight	1	0.021	0.887	1	5.953	0.024	1	0.187	0.670
Ratio of seed set	1	17.477	0.000	1	24.342	0.000	1	0.240	0.630
Ratio of tiller regeneration	1	6.157	0.015	1	3.375	0.069	1	4.255	0.042
F_3_ experiment									
Seed germination ratio-0 day	1	1.023	0.341	1	2.527	0.151	1	0.002	0.963
Seed germination ratio-20 days	1	70.621	0.000	1	79.724	0.000	1	1.103	0.324
Seed germination ratio-40 days	1	65.154	0.000	1	13.462	0.006	1	10.560	0.012
Seed germination ratio-60 days	1	21.437	0.002	1	35.438	0.000	1	6.509	0.034

Df, degree of freedom; F, F values; P, level of significance.

**Figure 2:**
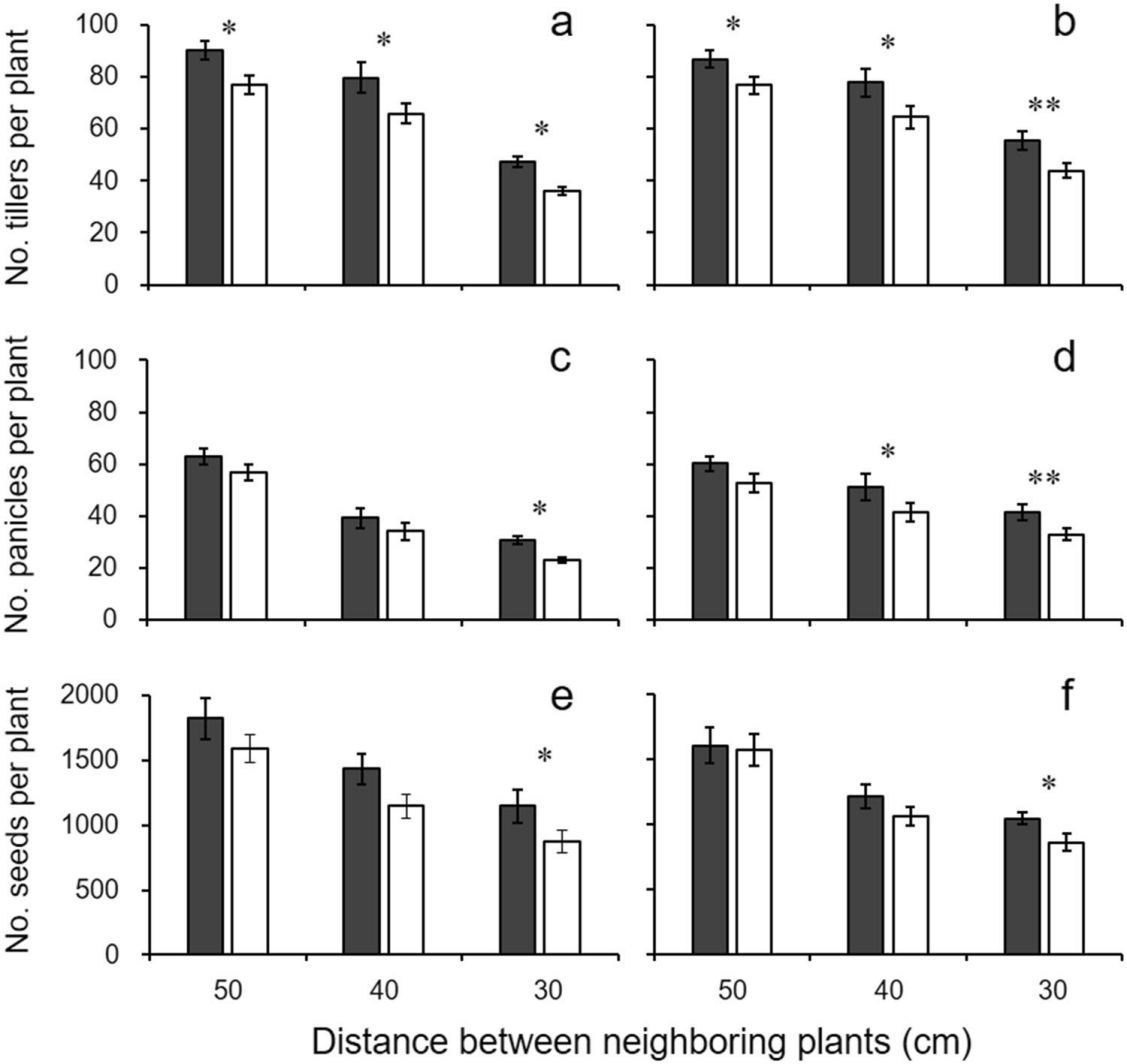
Number of tillers (**a**, **b**), panicles (**c**, **d**), and filled seeds (**e**, **f**) per plant in F_1_ transgenic (dark grey columns) and non-transgenic (white columns) crop-wild hybrids derived from WR1 (left panel) and WR2 (right panel), in mixed-planting plots (30 cm, 40 cm, 50 cm plant spacing) with different densities. The comparisons were made between transgenic and non-transgenic F_1_ hybrids in mix planting based on paired *t*-test (N = 6). Bars represent standard error. * or ** indicates significances at the levels of P < 0.05 or P < 0.01, respectively.

Two-way ANOVAs showed significant effects of a transgene (T) and wild parents (WR) on the number of tillers per plant (at different growth stages), days to flowering, and ratios of seed sets in both F_1_ and F_2_ hybrid descendants. However, no significant interaction effect was detected (Table 1). In the pure planting mode, significant increases were detected for the number of tillers per plant in F_1_ transgenic hybrids throughout the growth stages (with 19~38% increase); but significant increases were mainly detected at the early growth stages in F_2_ transgenic hybrid lineages (with 16~17% increase) (Fig. 1a and b; Table S1). In the mix-planting mode with 30 × 30 cm spacing between plants, significant differences were also detected for the number of tillers per plant, but with more prominent differences between transgenic and non-transgenic hybrid lineages (with 26~49% increase in F_1_ and 9~24% increase in F_2_) (Fig. 2a and b; Tables S2 and S3). Noticeably, the increase in number of tillers was more pronounced in the 30 × 30 cm mix-planting plot than that of other plots with lower densities (Fig. 2a and b; Tables S2 and S3). In addition, transgenic hybrid plants flowered significantly earlier (3 days for F_1_ and 9~15 days for F_2_) than their non-transgenic counterparts (Fig. 1c and d; Table S1). Consequently, transgenic hybrid plants had significantly higher ratios of seed sets (29% in F_1_ derived from WR1 and 29–30% in F_2_) than their non-transgenic counterparts in pure planting (Fig. 1e and f; Table S1). The ratios of seed sets were negatively correlated with the days to flowering in F_2_ hybrid lineages with or without the transgene (Fig. S1a and b), suggesting the possible contribution of earlier flowering to greater seed sets.

### Increased fecundity in transgenic hybrid lineages

**Figure 3:**
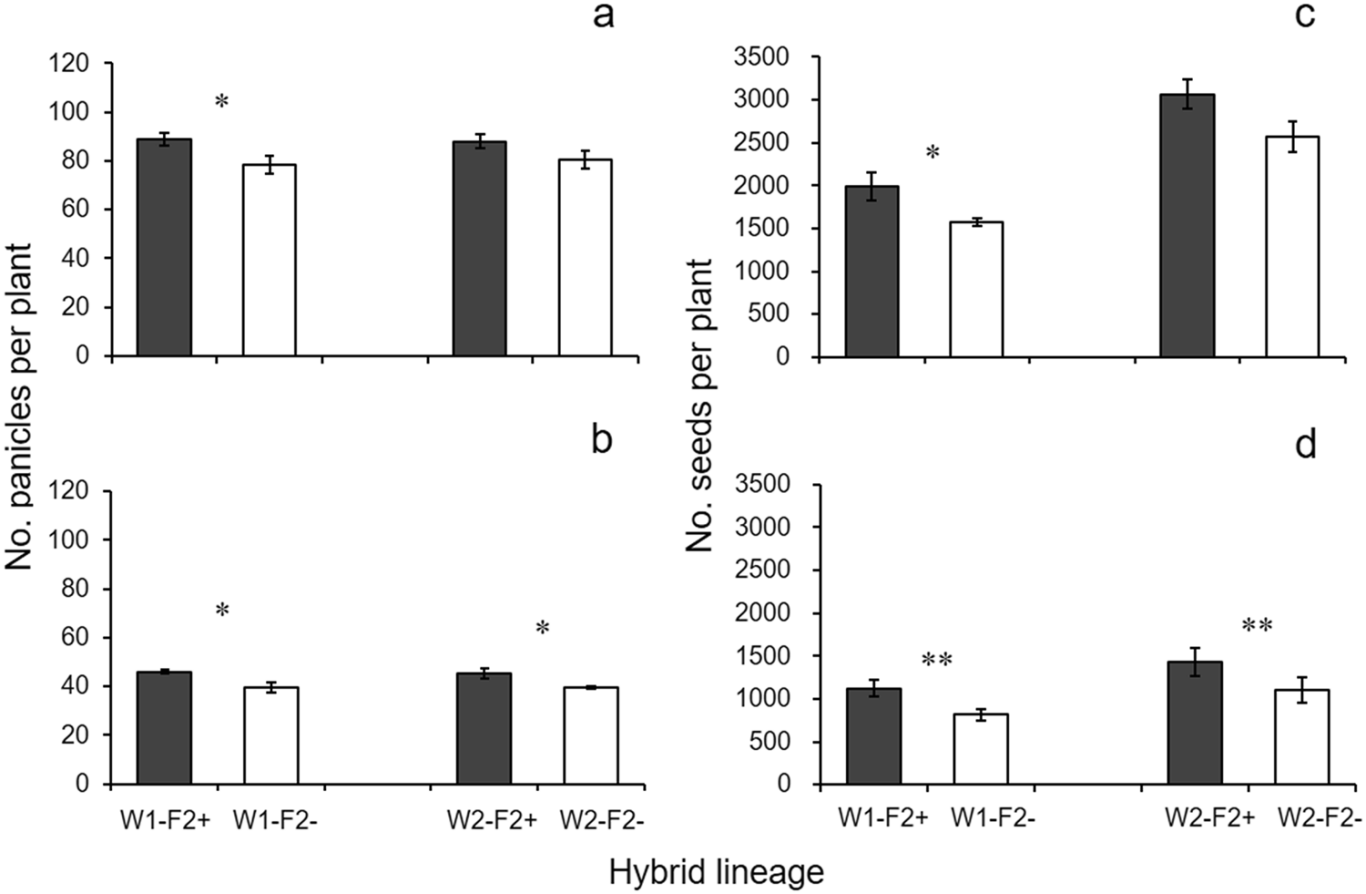
Number of panicles (**a**, **b**) and filled seeds (**c**, **d**) per plant of F_2_ transgenic (dark grey columns) and non-transgenic (white columns) crop-wild hybrid lineages in pure-planting (**a**, **c**) and mix-planting plots (**b**, **d**). The comparisons were made between transgenic and non-transgenic F_2_ hybrid lineages in mix planting (30 cm) based on paired *t*-test (N = 6). Bars represent standard error. * or ** indicates significances at the levels of P < 0.05 or P < 0.01, respectively.

Both F_1_ and F_2_ transgenic hybrid lineages showed significantly increased fecundity as indicated by the number of panicles and seeds per plant compared to their non-transgenic counterparts in mix-planting plots with 30 × 30 cm spacing (Figs 2c, d, e, f and 3b and d; Tables S2 and S3). The hybrid lineage with WR1 as the wild parent also showed significantly increased fecundity in pure planting (Fig. 3a and c; Table S1). In addition, no differences were detected for 1000-seed weight between transgenic and non-transgenic hybrid lineages (Table 1). Two-way ANOVAs showed a significant effect of transgene (T) on the number of panicles per plant in F_1_ and F_2_ crop-wild hybrid descendants (Table 1). Wild parent (WR) had significant effect on the number of seeds per plant in F_1_-F_2_ and 1000-seed weight in F_2_ hybrid descendants (Table 1). No significant interaction effect was detected (Table 1). In the pure planting mode, 17~22% (F_1_) and ~13% (F_2_) increases were detected in number of panicles per plant in transgenic hybrid lineages (Fig. 3a; Table S1); meanwhile, ~27% increase was detected for the number of filled seeds per plant in F_2_ transgenic hybrid lineages (Fig. 3c; Table S1). In the mix-planting mode with different densities, significant increase in number of panicles (with 10~33% increase) and filled seeds (with 21~38% increase) per plant was mainly observed in the 30 × 30 cm plots in F_1_ and F_2_ transgenic hybrid lineages (Figs 2c, d, e, f and 3b and d; Tables S2 and S3). It seems that with the increase in cultivation densities, the extent of increases in panicles and filled seeds per plant between transgenic and non-transgenic plants became more substantial, suggesting the competitive effect for fecundity (Fig. 2c, d, e, and f).

In addition, the two transgenic events (EP3 and EP4) showed similar extent of glyphosate resistance and increased number of panicles and filled seeds per plant (Table S4), suggesting the observed differences between transgenic and non-transgenic lineages were not the result of transgene insertion effect.

### Enhanced buried-seed germination and over-winter regeneration in transgenic hybrid lineages

**Figure 4:**
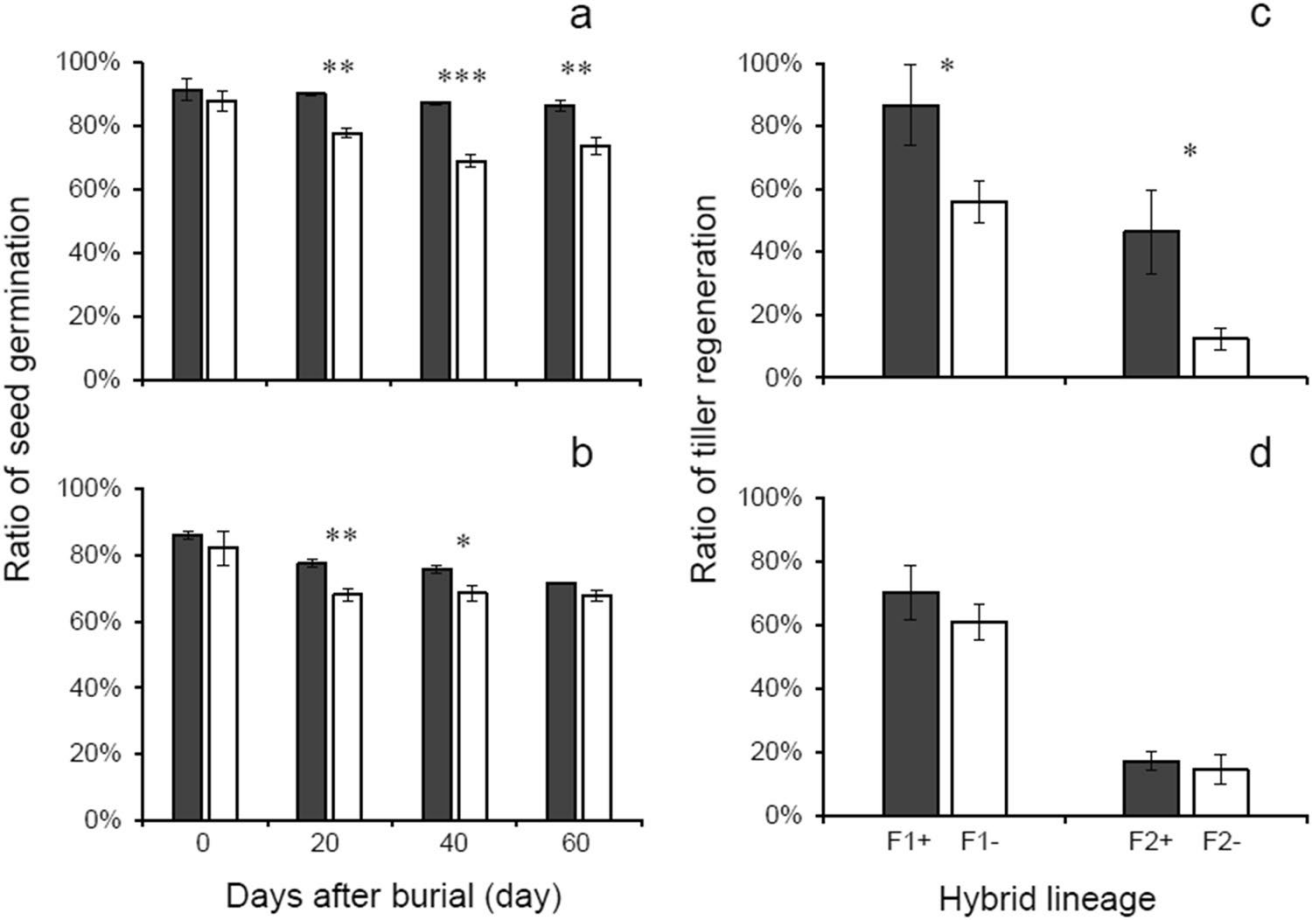
Germination ratios of buried-seeds (**a**: WR1-F3, **b**: WR2-F3) and ratios of tiller regeneration (**c**: WR1-F1 and WR1-F2, **d**: WR2-F1 and WR2-F2) in transgenic (dark grey columns) and non-transgenic (white columns) crop-wild hybrid lineages. The comparisons were made between transgenic and non-transgenic hybrid lineages based on independent *t*-test (N = 3 for seed germination ratio; N = 6 for tiller regeneration ratio). Bars represent standard error. * or ** indicates significances at the levels of P < 0.05 or P < 0.01, respectively.

The transgenic hybrid lineages had higher germination ratios for soil-buried seeds (F_3_) and tiller regeneration ratios overwinter (F_1_ and F_2_) than non-transgenic counterparts, especially for those derived from WR1 (Fig. 4a, b, c and d; Tables 1 and S1).

Two-way ANOVAs showed significant effect of transgene (T) on the ratios of seed germination (after being buried for 20, 40, and 60 days) and tiller regeneration. Wild parent (WR) had significant effect on ratios of buried-seed germination. Significant effect was detected for interaction between T and WR on the ratios of buried-seed germination (only for 40 and 60 days) and tiller regeneration (Table 1). No significant differences in germination ratios were detected between transgenic and non-transgenic hybrid lineages before seed burial. However, transgenic hybrid lineages (from WR1) showed 26%, 45%, and 38% higher seed germination ratio than their non-transgenic counterparts, 20, 40, and 60 after days after burial (Fig. 4a). A similar trend was also observed in hybrid descendants derived from WR2 (Fig. 4b). Transgenic F_1_ and F_2_ hybrid lineages derived from WR1 showed 55% and 275% higher tiller regeneration ratios compared to their non-transgenic counterparts (Fig. 4c). However, no significant differences were detected between hybrid lineages derived from WR2 (Fig. 4d), indicating apparent maternal influences.

### Differences in fitness-related traits affected by endogenous EPSPS level of transgene recipients

**Figure 5:**
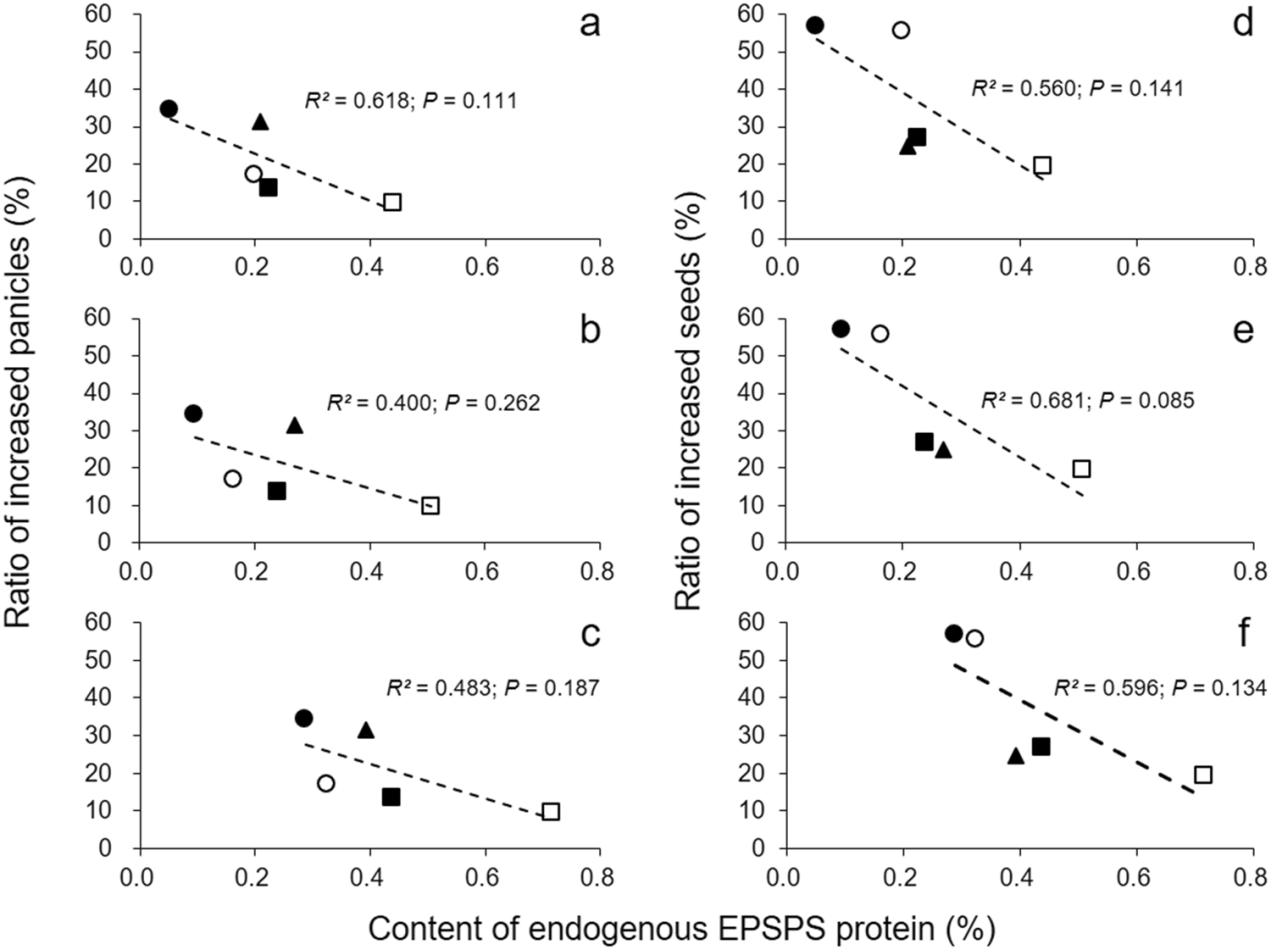
Correlation between the ratios of increased panicles (**a**, **b**, and **c**) or seeds (**d**, **e**, and **f**) and the content of endogenous EPSPS proteins in different transgene recipients (parents) at 60 (a and d), 100 (**b** and **e**), and 160 (**c** and **f**) days after seed germination. Solid diamonds: wild rice (WR1); empty diamonds: wild rice (WR2); empty circles: weedy rice (WRD1); solid circles: weedy rice (WRD2), and solid triangles: cultivated rice (Minghui-86).

The content of endogenous EPSPS was varied significantly among different transgene recipients (WR, WDR, and cultivated rice parents) at different growth stages (Fig. 5). The degree of fitness differences (as indicated by the ratios of increased panicles and seeds per plant) between transgenic and non-transgenic hybrid lineages varied substantially among different transgene recipients. A weak negative correlation was observed between the fitness differences caused by the incorporation of the *epsps* transgene and the content of endogenous EPSPS of different types of the transgene recipients (Fig. 5).

The two WDR populations expressed a low level of endogenous EPSPS (0.05~0.33%) at different growth stages. Accordingly, their transgenic lineages showed a more substantial increase in the number of panicles (17~34%) and filled seeds (55~57%) per plant (Fig. 5). In contrast, the two WR populations expressed a relatively high level of endogenous EPSPS (0.23~0.72%), and their transgenic counterparts showed a less increase in the number of panicles (9~13%) and filled seeds (19~27%) (Fig. 5). The parental rice line (Minghui-86) showed a moderate level of endogenous EPSPS and fecundity change (Fig. 5) compared to the WR and WDR populations. These results suggested the possible association, although not strong, between the levels of endogenous EPSPS expression and differences in fitness caused by the *epsps* transgene.

## Discussion

We found evidently altered phenological characteristics (e.g., higher tillering rates and earlier flowering), increased fecundity (more seeds and higher seed-set ratios), and enhanced ability of overwinter survival for stocks/tillers in the crop-wild rice hybrid lineages containing an *epsps* glyphosate-resistance transgene, based on our three-year common-garden experiments. These results suggest that the transgene over-expressing *epsps* can change the life-cycle characteristics and increase fitness of crop-wild rice hybrid descendants in the glyphosate-free environment, similar with that reported by Wang *et al*.^16^ in a study with the same *epsps* transgene in crop-weed hybrid descendants. In addition, we also detected differences in the base-line expression level of the EPSPS protein encoded by the endogenous *epsps* gene in transgenic recipients (parents), which influenced the fitness effect of the *epsps* transgene in their corresponding crop-wild/weed hybrid lineages. This result suggests that the genetic background of transgene recipient populations can considerably affect fitness of a transgene, which is similar with that in a study involving two insect-resistance transgenes (*Bt*, *Bt/CpTI*)^18^. Altogether, these findings answered our questions raised up earlier that crop-to-wild introgression of a glyphosate-resistance transgene overexpressing *epsps* through gene flow will significantly change the life-cycle fitness of WR populations in the herbicide-free habitats. In general, such altered life-cycle fitness of crop-wild hybrid descendants caused by the transfer of a transgene overexpressing *epsps* to wild relatives will likely generate unwanted environmental impact, including the creation of more invasive agricultural weeds and losses of genetic integrity of wild relative species of crops.

The higher tillering rates at different growth stages and earlier flowering time will likely increase the competitiveness of the hybrid plants that acquired the *epsps* transgene and shift their reproductive period some days ahead. The increased tillering rate detected in crop-wild hybrid lineages from this study is similar as that reported in the previous study based on analyses of crop-weed hybrid lineages^16^. Our findings suggest that transgenes overexpressing EPSPS may promote the persistence and spread of crop-wild and crop-weed transgenic hybrid descendants in rice in the glyphosate-free environment. However, some studies did not show fitness changes in mutational herbicide-resistant weeds with multiple copies of endogenous *epsps* genes^47–49^, suggesting the complex of the *epsps* genes in terms of the fitness change in plants. Further studies are required to reveal the underlying mechanisms. It is well known that EPSPS encoded by the *epsps* gene is a key enzyme in the shikimic acid pathway that is closely associated with plant growth through producing necessary aromatic amino acids, lignin, flavonoids, phenolics, and other secondary metabolic substances^50, 51^. The overexpressed EPSPS by transgene will not only improve the glyphosate-resistance of the transgene recipient^52^, but also affect their phenotype associated with the change in shikimic acid pathway. Probably, the observed higher tillering rates and earlier flowering of transgenic crop-wild hybrid lineages in our study was caused by the exceeded production of EPSPS that promoted the biosynthesis of metabolic substances in the downstream pathways^53, 54^. Consequently, the altered phenological characteristics advanced the reproductive period of transgenic hybrid plants, which may provide more opportunities for hybrid lineages to reproduce sexually under more favorable climate conditions (e.g., temperature and moistures) and to circumvent cold stress before winter comes. These changes resulted in the increases in seed production and seed-set ratios, as observed in our experiments. In addition, more vigorous plant growth as indicated by increased tillers can enhance the competitive ability of *epsps* transgenic hybrid plants, compared to those without the transgene. This hypothesis gains a support from the mix-planting plots with dense cultivation of plants in our experiments, where differences in the number of tillers, panicles, and filled seeds between transgenic and non-transgenic plants were more outstanding. The increased competitiveness and altered reproductive period of transgenic hybrid plants may eventually affect the life-cycle fitness of the WR populations.

The fecundity or seed production of hybrid lineages as indicated by the increased number of panicles and well-developed seeds per plant was considerably affected by the altered phenological characteristics as indicated above. Obviously, the greater number of panicles per plant observed in transgenic hybrid lineages is associated with their greater number of tillers per plants and enhanced competitiveness at the early growth stages. The increased number of seeds per plant found in transgenic crop-wild hybrid lineages is more likely attribute to both greater number of panicles per plant and the increased seed-set ratios, as affected by the advanced reproduction in slightly warmer conditions. The increased fecundity brought by the *epsps* transgene was also observed in other studies. For example, the *epsps* transgene increased fecundity of F_1_-F_2_ crop-weed hybrid lineages in rice^16^. The yield increase was also recorded in the GE glyphosate-resistant (CP4) soybean cultivars, compared with their non-GE counterparts in the absence of glyphosate^55^, although the authors attributed the observed yield increase to the improved genetic background for the GE cultivars. In addition, a study of GE glyphosate-resistant wheat lines containing an *epsps* (CP4) transgene driven by rice actin1 and 35S promoters showed considerable increases in the grain yield without glyphosate applications in an experiment^56^. All these studies demonstrate that the *epsps* transgene can increase fecundity of GE crops and particularly crop-wild/weed hybrid descendants in the environment without the application of glyphosate herbicides, which will promote the persistence and spread of the transgenes.

The significantly enhanced overwinter ability of transgenic hybrid descendants (particularly those derived from WR1), as measured by seed germination after being buried in the soil and tiller survival/regeneration through winters, indicates enhanced tolerances of hybrid plants to environmental stresses by introducing the *epsps* transgene. The increased germination ratios of transgenic seeds after being buried in soils is probably due to their increased tolerance to the biotic and abiotic stress in the soil, although the underlying mechanisms need further studies to be revealed. In addition, the enhanced ability of tiller survival/regeneration in transgenic hybrid lineages suggests their enhanced tolerance to cold stress in winter, promoting the initial competitive ability of transgenic hybrid seedlings to occupy more territories. WR populations can propagate vegetatively mainly through tillers/stocks and ratoons during the winter, particularly for the large populations occurring in deep-water habitats^57^. The overwintering survival/regeneration ability of the transgenic plants is critical for the successful maintenance and expanding of *O*. *rufipogon* populations in the next year. It is apparent that the enhanced ability of vegetative propagation through winter, together with the increased fecundity of crop-wild hybrid descendants containing the *epsps* transgene, will considerably increase the life-cycle fitness advantages, resulting in unwanted environmental consequences.

In addition, our results of significant differences in base-line endogenous EPSPS contents among different WR, WDR populations and cultivated rice line indicate the influences of genetic background of transgene recipients on the fitness effect. For example, the two WDR populations with a lower level of endogenous EPSPS showed much more pronounced enhancement in fitness (e.g., number of panicle and seeds per plant) in crop-weed hybrid descendants than two WR populations with a higher EPSPS level. Probably, WDR plants with relatively lower level of endogenous EPSPS response more sensitively to the *epsps* transgene, leading to more dramatic changes in fitness in transgenic crop-weed hybrid descendants, and vice versa. A similar phenomenon was also found in another study, in which different fitness effects of insect-resistance transgenes were detected in crop-weed hybrid descendants derived from crosses between a GE rice line and five WDR populations with different origins, respectively^18^. Therefore, differences in the genetic background should be considered in the biosafety assessment of transgene introgression into different wild/weedy recipient populations, following the case-by-case principle^2, 3, 5^.

In conclusion, our study demonstrated the altered phenology and enhanced life-cycle fitness of crop-wild hybrid descendants containing the *epsps* transgene in the glyphosate-free environment. The fitness change may cause unwanted environmental consequences, once this type of herbicide-resistance transgene introgressed into WR populations through gene flow. On one hand, WR individuals/populations that have picked up the *epsps* transgene may become more aggressive weeds because of their increased fitness benefit in natural habitats, although the problem may become more serious in the agricultural ecosystems where glyphosate herbicides are applied^1, 3, 6, 7, 23^. On the other hand, introgression of the *epsps* transgene may also affect the genetic integrity of local WR populations and cause their reduction or even extinction due to genetic swamping and selective sweep effects^1, 3, 43, 58^. Therefore, it is necessary to design proper strategies to effectively assess and manage the potential risks caused by introgression of transgenes that can increase weediness and invasiveness of wild relative populations.

## Materials and Methods

### Production of crop-wild hybrid lineages

An herbicide-resistant transgenic rice (*Oryza sativa* L.) line (EP3), its non-transgenic rice parent (Minghui-86), and two WR populations (WR1, WR2) were used to generate F_1_-F_3_ crop-wild hybrid descendants. The EP3 transgenic line containing a GE endogenous *epsps* gene from rice was produced *via* the *Agrobacterium*-mediated transformation from Minghui-86^16, 59^. The EP3 line used for hybridization was a T5 generation homozygous for the *epsps* transgene that was resistant to glyphosate^34^. The non-transgenic Minghui-86 is a widely-used rice variety in China. The two *O*. *rufipogon* populations were collected from Dongxiang in Jiangxi province (WR1) and Suixi in Guangdong province (WR2), China. To avoid the possible gene insertion effects at different loci on phenotypes, we analyzed glyphosate resistance and two fecundity-related traits (number of panicles and filled seeds per plant) of two independent transgenic rice lines (EP3 and EP4), using their non-transgenic parent (Minghui-86) as a control.

**Figure 6:**
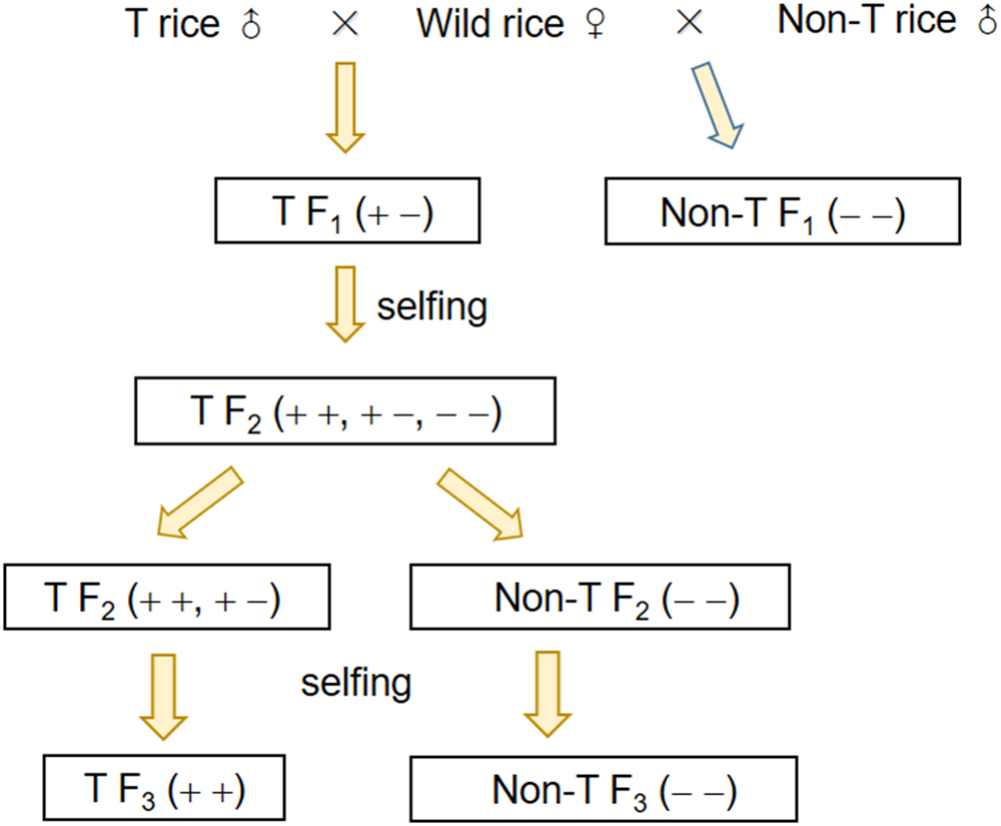
Schematic illustration of the pedigrees to produce F_1_-F_3_ crop-wild hybrid lineages. T: transgenic; + +, + −, and − −: transgene homozygous, transgene heterozygous, and non-transgenic, respectively. F_1_ and F_2_ hybrid lineages were used to test differences in fitness; the F_3_ hybrid lineages were used to test differences in seed germination after being buried in soils.

For creating crop-wild hybrid lineages, we produced F_1_ transgenic and non-transgenic hybrids by hand pollination of WR plants (more than 20 plants per population) with EP3 and Minghui-86 rice lines in the designated Biosafety Assessment Centers in Fuzhou, Fujian Province. We selfed the F_1_ transgenic hybrids (EP3 × WR) to produce F_2_ and F_3_ hybrid lineages that segregated for the presence and absence of the target transgene to estimate the fitness effect of *epsps* transgene under the same genetic background in advanced generations of crop-wild hybrid descendants. The F_2_ transgenic hybrid plants used in the field experiment contained either hemizygous or homozygous *epsps* transgene, whereas the F_3_ transgenic hybrid plants only contained homozygous *epsps* transgene (Fig. 6).

The identification of transgene status of crop-weed hybrid descendants in F_1_ and F_2_ generations was achieved by specific molecular markers^16^. For the F_3_ hybrids, we obtained transgene homozygous and non-transgene homozygous F_3_ hybrid lineages by randomly screening more than 15 seeds harvested from each F_2_ plant for the presence or absence of transgene. The above homozygous lineages were preserved for future F_3_ experiment. Thus, the plant materials used in the experiments included WR parents (WR1, WR2), F_1_ hybrids (WR1-F1+ and WR2-F1+ with presence of *epsps* transgene; WR1-F1- and WR2-F1- with absence of *epsps* transgene), F_2_ hybrid lineages (WR1-F2+ and WR2-F2+ with presence of *epsps* transgene; WR1-F2- and WR2-F2- with absence of *epsps* transgene), and F_3_ hybrid lineages (WR1-F3+ and WR2-F3+ with presence of *epsps* transgene; WR1-F3- and WR2-F3- with absence of *epsps* transgene).

### Field experiment design

Common garden field experiments were carried out in the designated Biosafety Assessment Centers in Fuzhou, Fujian Province, to estimate the effects of *epsps* transgene on vegetative growth, phenology, fecundity, and overwintering regeneration. Six sets of materials were included in the experiments: transgenic F_1_ or F_2_ hybrid lineages and their non-transgenic F_1_ or F_2_ counterparts derived from WR1 and WR2, as well as the two wild parents. Two cultivation modes, pure planting of transgenic, non-transgenic hybrid lineages, or the parents and mix planting of transgenic and non-transgenic hybrid lineages alternately at different densities, were designed to estimate the competitive abilities between the transgenic and non-transgenic hybrid lineages. For each treatment, six replicates (plots) were included. In pure planting, each plot included 36 plants in 6 × 6 grid with 50 cm spacing for the F_1_ and F_2_ experiments. In mix planting, each plot also included 36 plants in 6 × 6 grid but with 30 cm spacing for the F_1_ and F_2_ experiments, and 40 cm and 50 cm spacing only for the F_1_ experiment. Consequently, a total of 72 (in F_1_) or 48 (in F_2_) plots were included for the field experiments. The field layout of all experimental plots followed a complete randomized design.

Seed burial experiments were carried out in the confined experimental blocks of Fudan University campus in Shanghai to estimate the germination ability of hybrid seeds after being buried in soils. Four groups of F_3_ hybrid seeds were included for the seed burial experiments: WR1-F3+, WR1-F3-, WR2-F3+ and WR2-F3-. These hybrid seeds were treated at 50 °C for 7 days to break the seed dormancy. The treated seeds were buried in the soil of a rice field after rice harvesting, for 0, 20, 40, and 60 days before seed germination. Consequently, a total of 16 treatments with three replicates (bags) and 48 bags were included in the experiments. Each nylon bag contained 50 seeds that was randomly buried in 10 cm depth of soils from December to next-year February. The buried seeds were moved out at the different days after burial and germinated on the moist filter papers in a petri dish at 30 °C to examine the seed germination (see detail in Table S5).

### Correlation between endogenous EPSPS contents in parental plants and fitness change caused by the transgene

To study relationships between endogenous EPSPS protein contents and fitness changes, we measure EPSPS contents in transgene recipient parents, including WR (WR1 and WR2), WDR (WDR1 and WDR2), and cultivated rice (Minghui-86), using ELISA (enzyme linked immunosorbent assay), in addition to the increased panicles and seeds per plant. The EPSPS protein content was measured as the ratio (%) between the amount of EPSPS protein and the total amount of soluble proteins. Pooled leaf tissues from three plants were collected as one sample (replicate) and nine samples from each type of parental plants were included at the vegetative (60 days), reproductive (100 days), and ripening stages (160 days). The Quantiplate kit (Envirologix, Portland, OR, USA) was used for the detection of the EPSPS proteins following the ELISA manufacturer’s protocols. We set the wavelength of the microtiter plate reader to 450 nanometers (nm) using Plate Reader (Bio-Rad Laboratories, Inc., Hercules, CA, USA). The MICROPLATE MANAGER (MPM) software ver. 6 (Bio-Rad Laboratories, Inc.) was used to summarize the results. The ratios of increased number of panicles and seeds per plant were estimated between transgenic and non-transgenic crop-wild or crop-weed hybrid lineages in the F_2_ generation, and between EP3 and Minghui-83. Data of crop-weed hybrid lineages used in this study were from Wang *et al*. (2014).

### Data collection and analysis

The methods for data collection follows the description in Table S5. Two-way ANOVAs were carried out to analyze the effects of transgene (transgenic *vs*. non-transgenic), wild parent (WR1 *vs*. WR2), and their interaction on fitness in pure-planting plots. Independent and paired *t*-tests were used to determine differences between transgenic and non-transgenic hybrid lineages for fitness-related traits in pure-planting pots and mix-planting plots, respectively. Independent *t*-tests was used to detect differences in endogenous EPSPS contents between WR1 and WR2 based on the ELISA experiment. The correlation between endogenous EPSPS protein contents and fitness changes was calculated based on Pearson Correlation Coefficient. All statistical analyses were performed using the software IBM SPSS Statistics ver. 22.0 for Windows (SPSS Inc., IBM Company Chicago, IL, USA, 2010).

### Data Availability

The datasets generated during and/or analysed during the current study are available from the corresponding author on reasonable request.

## Electronic supplementary material

Supplementary Information
