# Supplementary material for: Genetically engineered rice endogenous 5-enolpyruvoylshikimate-3-phosphate synthase (epsps) transgene alters phenology and fitness of crop-wild hybrid offspring
Source: Sci Rep. 2017 Jul 28;7:6834. doi: 10.1038/s41598-017-07089-9 (PMC5533792; doi:10.1038/s41598-017-07089-9)
Supplement: Supplementary file 1 — Supplementary Information [file 41598_2017_7089_MOESM1_ESM.pdf]

Genetically engineered rice endogenous 5-enolpyruvoylshikimate-3-phosphate synthase (*epsps*) transgene alters phenology and fitness of crop-wild hybrid offspring

Xiao Yang[1], Lei Li[1], Xiaoqi Jiang[1], Wei Wang[1], Xingxing Cai[1], Jun Su[2], Feng Wang[2], Bao-Rong Lu[1*]

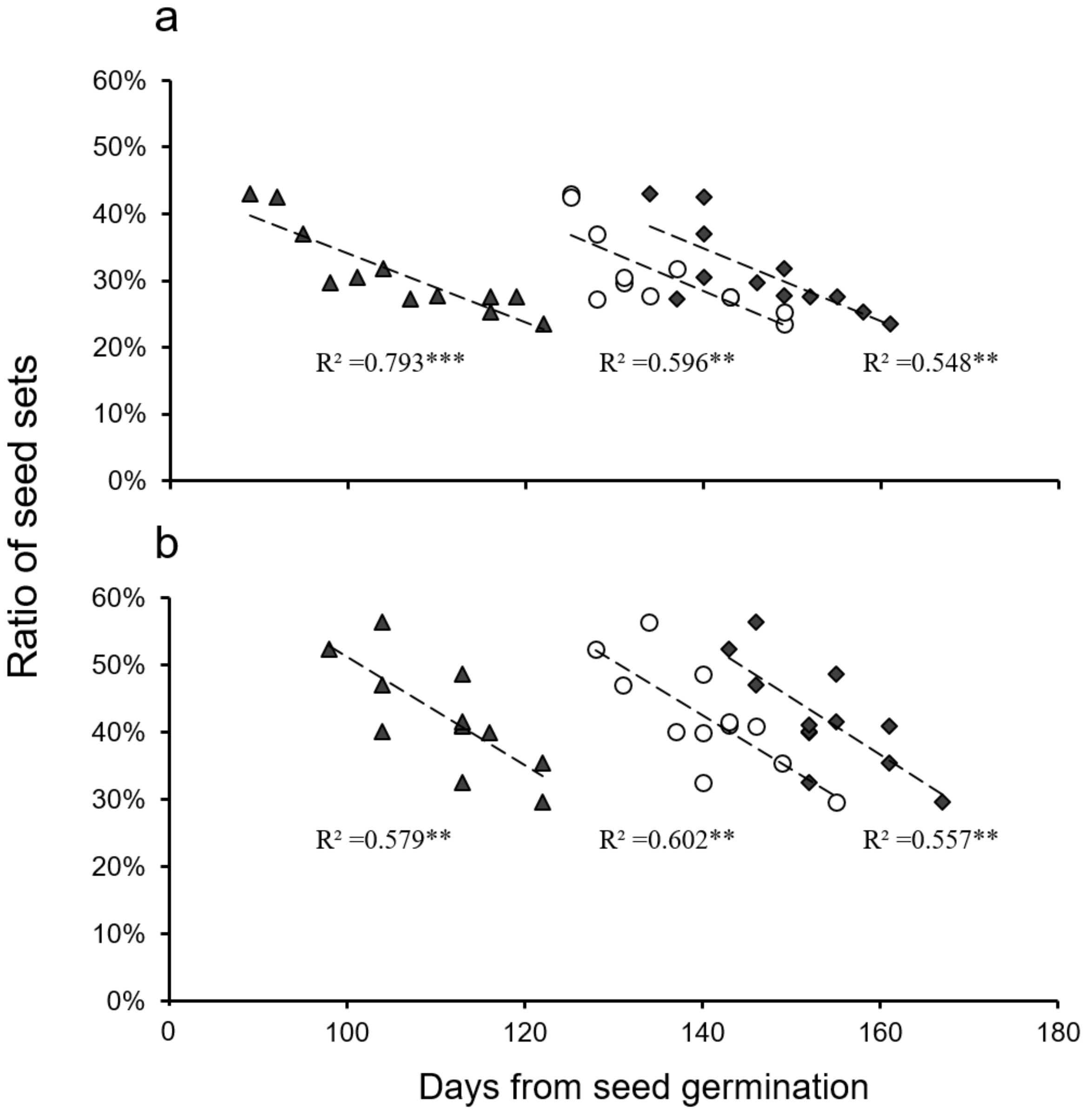

Figure S1. Correlations between seed-set ratios and days at which 1%, (triangles), 30% (circles) and 50% (diamonds) plants being flowered in WR1-F2 (a) and WR2-F2 (b) rice crop-wild hybrid lineages. R value was calculated based on Pearson Correlation Coefficient, ** or *** indicates significances at the levels of $P<0.01$ or $P<0.001$.

Table S1. Average values and standard errors (±) of life-cycle fitness-related traits of the wild rice (*Oryza rufipogon*) parent, F_1-F_2 transgenic and non-transgenic crop-wild hybrid lineages in pure-planting. Comparisons were made between transgenic and non-transgenic hybrid lineages based on the independent *t*-test (N=6). *, ** or *** indicates significances at the levels of P<0.05, P<0.01 or P<0.001.

Trait	WR1 combination			WR2 combination		
	WR1 parent	Transgenic lineage	Non-transgenic lineage	WR2 parent	Transgenic lineage	Non-transgenic lineage
**F1 experiment**						
Plant height (cm)	137.1±2.9	169.4±2.8	168.8±5.2	134.5±1.6	168.0±5.6	171.3±5.8
No. tillers per plant-30 days	9.8±0.4	30.1±2.2	21.8±1.6 *	13.3±0.6	23.6±1.0	18.2±0.6 ***
No. tillers per plant-60 days	65.0±3.4	68.8±2.0	54.8±2.0 ***	65.0±4.0	66.5±3.0	55.9±2.4 *
No. tillers per plant-150 days	80.7±6.0	86.0±2.5	68.5±2.4 ***	82.1±3.6	83.2±3.8	69.8±3.0 *
Days for 1% plants to flower	173.7±0.3	153.0±1.0	156.0±0.6 *	176.5±0.4	154.5±1.4	157.5±1.2
Days for 30% plants to flower	176.7±0.3	158.0±1.3	161.5±0.9	179.7±0.3	161.5±1.2	164.0±1.1
Days for 50% plants to flower	177.0±0.4	160.5±1.6	163.5±0.9	180.2±0.3	165.0±1.5	168.0±0.6
No. panicles per plant	63.0±3.7	55.6±3.4	47.7±1.6	70.9±6.7	59.6±4.0	48.9±2.8
No. seeds per plant	640.0±67.0	1817.4±149.7	1499.9±143.6	815.6±96.9	1288.1±113.4	1125.3±96.0
1000-seed weight (g)	16.5±0.1	20.8±0.2	21.3±0.4	14.9±0.1	20.7±0.4	20.7±0.2
Ratio of seed set (%)	66.7±4.3	34.0±1.3	26.4±1.8 **	63.4±3.1	40.7±1.7	39.9±2.0
Ratio of tiller regeneration (%)	224.7±28.9	86.7±12.9	56.0±6.7 *	254.6±20.1	70.4±8.6	60.9±5.8
**F2 experiment**						
Plant height (cm)	84.7±3.0	131.9±1.1	132.4±5.9	90.4±6.6	138.7±3.2	144.1±3.4
No. tillers per plant-30 days	16.0±0.8	29.1±0.9	24.9±1.4 *	18.9±1.0	28.2±0.9	24.3±1.0 *
No. tillers per plant-60 days	36.6±3.2	85.4±4.4	77.2±5.9	51.5±7.6	84.5±4.4	76.0±3.3
No. tillers per plant-150 days	50.4±4.4	103.4±2.9	89.4±4.0 *	71.7±8.0	94.7±2.5	88.7±3.5
Days for 1% plants to flower	169.2±0.3	97.5±2.8	114.0±3.1 **	172.2±0.3	106.5±2.7	116.0±1.9 *
Days for 30% plants to flower	173.2±0.3	129.0±1.8	141.5±3.1 **	175.2±0.3	135.0±2.0	146.0±2.2 **
Days for 50% plants to flower	174.0±0.3	141.0±2.3	152.5±3.0 *	175.8±0.3	149.0±1.9	158.0±2.4 *
No. panicles per plant	22.7±2.5	89.0±2.6	78.5±3.7 *	40.8±6.2	88.0±2.8	80.4±3.8
No. seeds per plant	253.0±35.7	1991.8±167.7	1571.1±49.0 *	430.2±68.4	3067.3±171.1	2568.0±180.3
1000-seed weight (g)	17.6±0.5	20.5±0.3	20.7±0.4	15.1±0.4	19.8±0.3	19.7±0.4
Ratio of seed set (%)	48.5±1.9	35.2±2.7	27.0±1.0 *	44.1±3.3	47.4±2.7	36.8±2.1 b *
Ratio of tiller regeneration (%)	189.8±15.5	46.4±13.4	12.4±3.5 *	228.7±20.2	17.2±2.8	14.5±4.7

Table S2. Average values and standard errors (±) of life-cycle fitness-related traits of F_1 transgenic and non-transgenic crop-wild hybrid lineages of rice in mix-planting with different densities. Comparisons were made between transgenic and non-transgenic hybrid lineages based on the paired *t*-test (N=6). * or ** indicates significances at the levels of P<0.05 or P<0.01.

Trait	WR1 combination		WR2 combination	
	Transgenic lineage	Non-transgenic lineage	Transgenic lineage	Non-transgenic lineage
**50cm spacing**				
Plant height (cm)	192.4±2.7	189.8±1.5	178.0±4.2	181.9±5.2
No. tillers per plant-30 days	28.3±2.0	22.4±1.8 *	22.0±1.5	16.2±1.0 *
No. tillers per plant-60 days	84.2±2.8	75.6±4.1 *	85.5±5.0	71.9±3.4
No. tillers per plant-150 days	90.1±3.7	76.8±3.6 *	86.8±3.4	76.8±3.5 *
No. panicles per plant	62.8±3.0	56.9±3.0	60.2±2.8	52.7±3.7
No. seeds per plant	1820.6±157.6	1585.6±108.2	1604.6±134.9	1572.2±125.2
1000-seed weight (g)	21.7±0.2	21.4±0.2	21.5±0.3	20.6±0.4 *
Ratio of seed set (%)	46.5±3.4	42.8±2.0	36.4±2.0	36.7±2.6
**40cm spacing**				
Plant height (cm)	180.9±4.5	178.5±5.5	172.2±4.9	165.3±2.9
No. tillers per plant-30 days	26.1±1.8	18.3±1.3 *	21.4±2.0	14.5±1.2 **
No. tillers per plant-60 days	66.5±2.8	59.4±2.0 *	70.7±6.4	53.2±5.2 *
No. tillers per plant-150 days	79.7±5.8	65.9±3.7 *	77.8±5.2	64.5±4.2 *
No. panicles per plant	39.2±3.9	34.0±3.1	51.0±5.2	41.4±3.5 *
No. seeds per plant	1431.1±117.5	1145.4±91.8	1213.8±89.2	1061.1±71.3
1000-seed weight (g)	21.7±0.2	21.5±0.3	21.1±0.4	21.0±0.4
Ratio of seed set (%)	45.0±2.3	42.5±2.2	35.6±2.7	34.7±1.5
**30cm spacing**				
Plant height (cm)	167.4±3.8	166.6±2.7	171.0±2.2	166.9±2.3
No. tillers per plant-30 days	22.9±2.4	15.4±0.5 *	19.1±1.5	14.1±1.1 **
No. tillers per plant-60 days	45.2±4.4	31.2±2.3 *	45.2±4.0	33.9±2.6 **
No. tillers per plant-150 days	47.3±2.0	36.3±1.5 *	55.4±3.7	44.0±3.0 **
No. panicles per plant	30.7±1.5	23.2±1.1 *	41.2±3.1	32.9±2.2 **
No. seeds per plant	1146.6±125.5	874.5±89.3 *	1046.0±48.4	863.5±65.2 **
1000-seed weight (g)	21.6±0.2	21.8±0.2	21.5±0.2	21.4±0.4
Ratio of seed set (%)	40.2±0.8	36.5±2.2	50.8±4.0	50.7±2.8

.

Table S3. Average values and standard errors (±) of life-cycle fitness-related traits of F_2 transgenic and non-transgenic crop-wild hybrid lineages of rice in mix-planting (density of 30cm × 30cm). Comparisons were made between transgenic and non-transgenic hybrid lineages based on the paired *t*-test (N=6). * or ** indicates significances at the levels of $P<0.05$ or $P<0.01$.

Trait	WR1 combination		WR2 combination	
	Transgenic lineage	Non-transgenic lineage	Transgenic lineage	Non-transgenic lineage
Plant height (cm)	127.5±4.7	124.4±4.1	127.8±1.7	121.3±6.9
No. tillers per plant-30 days	26.3±0.5	24.1±0.4 *	25.4±1.7	23.3±1.4 *
No. tillers per plant-60 days	37.8±1.2	30.4±1.1 **	37.1±2.8	30.6±1.3 *
No. tillers per plant-150 days	52.6±1.5	45.8±1.1 **	50.2±2.5	43.5±0.9 *
No. panicles per plant	46.0±0.7	39.6±2.0 *	45.2±2.1	39.4±0.4 *
No. seeds per plant	1124.7±90.3	814.3±72.3 **	1424.8±163.2	1105.0±145.5 **
1000-seed weight (g)	20.4±0.2	20.6±0.5	19.6±0.7	19.0±0.3
Ratio of seed set (%)	34.5±3.2	28.0±2.5 *	39.8±3.0	30.6±4.0 *

Table S4. Glyphosate-resistance (based on ED50 estimates), content of endogenous EPSPS protein and performance of yield-related traits of two transgenic rice lines (EP3 and EP4) and their parental rice line (Minghui-86). Seedlings at three-leaf stage were used for the testing of glyphosate-resistance. Mean±SE was shown for fecundity-related traits (N=5 plots). Comparisons were made among different rice lines for yield-related traits using Duncan's multiple range test. Groups that do not share letters are significantly different at $P<0.05$.

Rice line	LD50 (mmol/L)	EPSPS protein (%)	No. of panicles	No. of filled seeds
EP3	17.00	0.60~0.61	9.2±0.4 a	774.8±42.5 a
EP4	8.90	--	8.2±0.2 ab	730.4±32.6 a
Minghui-86	4.38	0.21~0.39	7.0±0.3 b	620.8±9.1 b

Table S5. Phenological and fitness-related characters measured in the common-garden field experiments.

Trait measured	Method of measurement
Plant height (cm)	Height from the base (ground) of a plant to the leaf tip of main tiller measured at maturity
No. tillers per plant	Tiller number of a plant that measured three times at 30, 60, and 150 days after transplanting
Days for different present of plants to flower (day)	Number of days from seed germination to flowering was recorded for each plant in pure-planting, then the days at which 1%, 30%, and 50% plants in a plot being flowered was calculated
No. of panicles per plant	Panicles number of a plant measured at panicle maturity
No. of seeds per plant	Number of well-developed (filled) seeds of a plant measured at panicle maturity
1000-seed weight (g)	Weight in grams of 1000 filled seeds measured based on sampling and weighing 100 randomly selected seeds three times from the same plant
Ratio of seed set (%)	Ratio between the number of filled seeds and the total number of spikelets harvested from a plant
Ratio of buried-seed germination (%)	Seeds token out from soil were soaked in moist filter papers in a petri dish and placed in oven with 30 Celsius degrees for two days; then the seeds were moved out to measure the seed germination ratio in each bag
Ratio of tiller regeneration (%)	Ratio between the number of early-regenerated tillers in second year and the total number of final tillers of a plant in the first year. The number of early-regenerated tillers per plant was measured in early March of each experimental year in pure planting plots
